# Comparative transcriptomics of female and male gametocytes in *Plasmodium berghei* and the evolution of sex in alveolates

**DOI:** 10.1186/s12864-017-4100-0

**Published:** 2017-09-18

**Authors:** Lee M. Yeoh, Christopher D. Goodman, Vanessa Mollard, Geoffrey I. McFadden, Stuart A. Ralph

**Affiliations:** 1 Department of Biochemistry and Molecular Biology, Bio21 Molecular Science and Biotechnology Institute, The University of Melbourne, Parkville, 3010 Australia; 2 School of BioSciences, The University of Melbourne, Parkville, 3010 Australia

**Keywords:** Plasmodium, Plasmodium berghei, Malaria, Gametocyte, Transcriptome, RNA-seq, Next-generation sequencing, Sex, Evolution

## Abstract

**Background:**

The clinical symptoms of malaria are caused by the asexual replication of *Plasmodium* parasites in the blood of the vertebrate host. To spread to new hosts, however, the malaria parasite must differentiate into sexual forms, termed gametocytes, which are ingested by a mosquito vector. Sexual differentiation produces either female or male gametocytes, and involves significant morphological and biochemical changes. These transformations prepare gametocytes for the rapid progression to gamete formation and fertilisation, which occur within 20 min of ingestion. Here we present the transcriptomes of asexual, female, and male gametocytes in *P. berghei*, and a comprehensive statistically-based differential-expression analysis of the transcriptional changes that underpin this sexual differentiation.

**Results:**

RNA-seq analysis revealed numerous differences in the transcriptomes of female and male gametocytes compared to asexual stages. Overall, there is net downregulation of transcripts in gametocytes compared to asexual stages, with this trend more marked in female gametocytes. Our analysis identified transcriptional changes in previously-characterised gametocyte-specific pathways, which validated our approach. We also detected many previously-unreported female- and male-specific pathways and genes. Transcriptional biases in stage and gender were then used to investigate sex-specificity and sexual dimorphism of *Plasmodium* in an evolutionary context. Sex-related gene expression is well conserved between *Plasmodium* species, but relatively poorly conserved in related organisms outside this genus. This pattern of conservation is most evident in genes necessary for both male and female gametocyte formation. However, this trend is less pronounced for male-specific genes, which are more highly conserved outside the genus than genes specific to female development.

**Conclusions:**

We characterised the transcriptional changes that are integral to the development of the female and male sexual forms of *Plasmodium*. These differential-expression patterns provide a vital insight into understanding the gender-specific characteristics of this essential stage that is the primary target for treatments that block parasite transmission. Our results also offer insight into the evolution of sex genes through Alveolata, and suggest that many *Plasmodium* sex genes evolved within the genus. We further hypothesise that male gametocytes co-opted pre-existing cellular machinery in their evolutionary history, whereas female gametocytes evolved more through the development of novel, parasite-specific pathways.

**Electronic supplementary material:**

The online version of this article (doi:10.1186/s12864-017-4100-0) contains supplementary material, which is available to authorized users.

## Background

*Plasmodium* infections alternate between a vertebrate host, where they cause malaria, and almost invariably a definitive mosquito host, where sexual reproduction occurs [1, 2]. The parasite genus includes *Plasmodium falciparum* and *P. vivax*, which infect human hosts, and *P. berghei*, which infects rodents, and is commonly used as an animal model. The symptomatic vertebrate stage of malaria is the asexual cycle, when parasites undergo multiple cycles of replication, lysis and re-invasion of erythrocytes. A small proportion of asexual parasites irreversibly differentiate into sexual gametocyte stages, which are the progenitors of gametes, and can be either female or male.

Gametocyte differentiation is regulated by transcription factors AP2-G (Apetala 2–gametocyte) and AP2-G2 (Apetala 2–gametocyte 2), with the former essential for development of asexual stages into gametocytes [3]. The time taken for this transformation into gametocytes varies widely by species, but in *P. berghei* it is completed in around 28 h [4].

Female gametocytes are considered temporarily dormant [5]; they possess an inactive pool of mRNA transcripts bound by a protein complex that includes DOZI (development of zygote inhibited) and CITH (CAR-I/Trailer Hitch homolog) [6, 7]. Ablation of DOZI in female gametocytes prevents zygote maturation [6]. Despite their apparent dormancy, female gametocytes possess an abundance of ribosomes and endoplasmic reticulum (ER), presumably in preparation for downstream fertilisation and other stages [5].

Male gametocytes exist for the sole task of producing short-lived male gametes [5]. They possess few ribosomes and a meagre amount of ER, suggesting a reduction in translation [5]. While male gametocytes also possess DOZI, this is present in lower levels than in females [8]. Correspondingly, ablation of DOZI has no effect on male viability [6].

Once mature male gametocytes are formed, they are capable of undergoing exflagellation into male gametes. This is triggered by exogenous factors associated with the change of environment within the mosquito digestive system, including xanthurenic acid, a change in pH and a reduction in temperature [9]. Exflagellation is preceded by a rapid eight-fold replication of DNA [10], which is followed by the extrusion of eight highly motile gametes, all within 10–12 min [5]. Activation of female gametocytes also occurs, causing them to egress from the host cell [5].

After conversion of haploid gametocytes into haploid gametes within the midgut of the mosquito, fertilisation occurs within hours; male and female gametes fuse, followed by movement of the nucleus of the male gamete into the female cell [5]. The diploid zygote subsequently develops into a motile ookinete within the mosquito midgut [11].

Gametocytes are thus the pivotal stage for transfer of parasites from vertebrate host to mosquito vectors. Blocking gametocytes would interrupt transmission and may be one element in the strategy to eradicate malaria. Despite this, there have been only limited high-throughput data that aim to dissect the gender-specific traits of *Plasmodium* gametocytes.

Three proteomic studies on separated female and male gametocytes have been published, one in *P. berghei* [8] and two in *P. falciparum* [12, 13]. Two of these studies compared female and male gametocytes to the precursor asexual stages, allowing identification of female- and male-upregulated proteins [8, 12]. There has been one previous transcriptomic analysis on separated female and male gametocytes, in *P. falciparum*, where proteomic data was also generated [13]. While this provides an excellent initial survey of transcripts present in these life stages, the analysis was not based on biological replicates, precluding statistical inferences of differential expression. In addition, asexual stages were not sequenced, which makes it impossible to identify gametocyte-specific genes.

We present here the complete transcriptomic analysis of separated female and male gametocytes in *P. berghei*. We have sequenced both female and male gametocytes, as well as the asexual stages from which gametocytes are derived. We identified numerous female- and male-specific genes in *P. berghei*, some of these validated by previous proteomic results, and some novel genes. Finally, these expression data have helped to clarify the evolution of sex in *Plasmodium* compared to other related organisms.

## Results

### Purification of female and male gametocytes

**Fig. 1:**
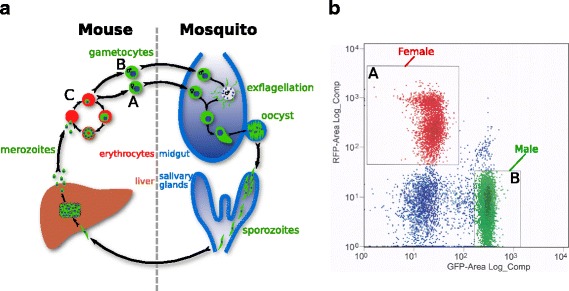
Purification of different stages of *P. berghei*. **a** Life-cycle schematic highlighting female gametocytes (A), male gametocytes (B), and asexual stages (C). **b** Fluorescence-activated flow cytometry plot showing collected samples of RFP female gametocytes (A) and GFP male gametocytes (B)

To purify female and male *P. berghei* gametocytes (Fig. 1 a), we used the previously-published parasite line 820cl1m1cl1 [14]. These parasites express either red fluorescent protein (RFP) or green fluorescent protein (GFP) under the control of the female- and male-specific promoters *ccp2* (PBANKA_1319500) and the dynein heavy chain (PBANKA_0416100) respectively. Samples were initially enriched for gametocytes by differential centrifugation. These stages were then sorted into RFP-expressing female gametocytes and GFP-expressing male gametocytes by flow cytometry (Fig. 1 b), and RNA extracted.

As a control, general intra-erythrocytic stages were also collected from infected mouse blood (Fig. 1 a), depleted of host RNA. These samples were not collected by flow cytometry, whereas it was necessary to collect gametocytes via this method; this difference in preparation might plausibly introduce some non-biological variation to detected expression differences. These samples are subsequently referred to as “asexual” stages in this manuscript. In addition to the asexual stages, these samples contained a minor pool of gametocytes, approximately 10% of total parasites, as assayed by microscopy. However, these impurities should only reduce the statistical power of our analyses, increasing the number of false negatives. Transcripts identified as gametocyte-specific below are in comparison to asexual stages obtained by cardiac bleed of infected mice. The use of this asexual population is consistent with previous studies [8], but we would expect a depletion of schizonts in this parasite sample [15], which could influence the genes that are identified as gametocyte-specific. Hence, our categorisation should be viewed in this context.

All stages were collected in biological triplicate to permit statistical analysis, with cDNA libraries enriched for poly-A transcripts, then sequenced on the Illumina platform.

### Differential expression analysis

**Fig. 2:**
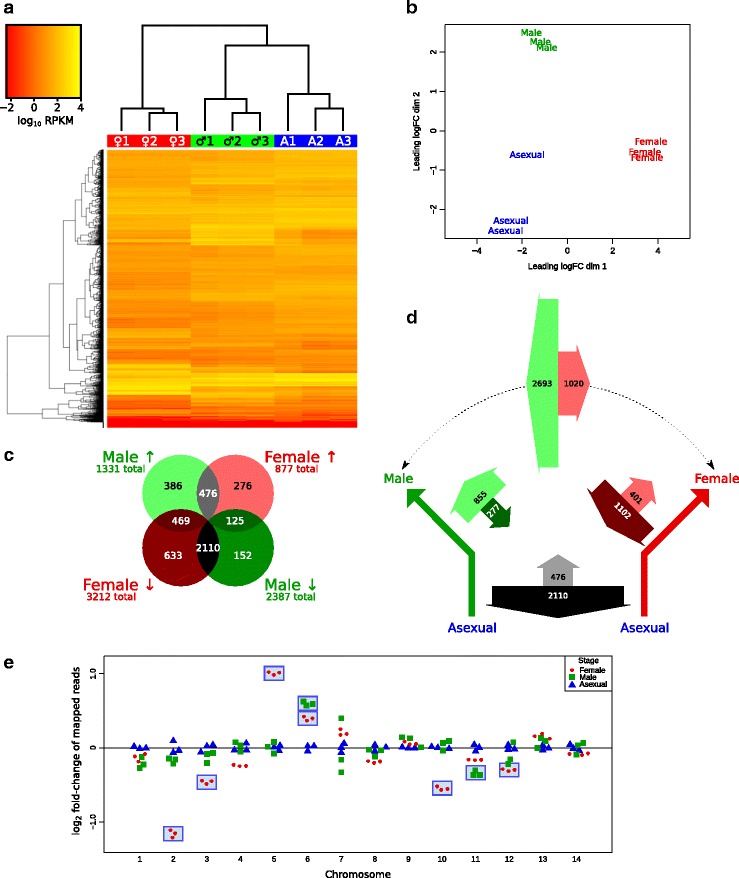
Comparative expression data for different stages. **a** Heatmap of expression, with genes on the vertical axis, and strains (male, ♂; female, ♀; asexual, A) on the horizontal axis. Replicates from each life-stage cluster independently (upper dendrogram). **b** Multi-dimensional scaling plot, showing clustering of sexual stages. **c** Venn diagram showing male and female genes that are differentially expressed compared to asexual genes, and the overlap between these groups. **d** Schematic depicting the asexual developmental precursor, and the differentiation into either female or male gametocytes. Thick arrows represent up- or downregulation, labelled with how many genes are affected, and arrow width proportional. Monochrome arrows represent shared up- or downregulation; red and green arrow pairs represent up- or downregulation specific to female or male gametocytes respectively. The green-red arrow pair on top represents genes identified by direct comparison of the two genders. **e** Fold-change of reads mapped to each chromosome compared to asexual samples. Each point represents a biological sample. The numbers of mapped reads were normalised to total reads mapped for that sample, then fold-change to the mean of the asexual counts for each chromosome plotted. Female and male samples with significant differences to asexual stages are marked by blue rectangles (*p* value < 0.05)

Samples were analysed for expression of genes. Clustering of samples based on expression of genes was assessed via a heatmap (Fig. 2 a; normalised read counts provided in Additional file 1) and multi-dimensional scaling plot (Fig. 2 b). In both cases, each set of replicates clustered to the exclusion of other stages. This indicates that biological and experimental variation between replicates is relatively minor compared to variation between distinct stages.

Female and male gametocytes were analysed for differential expression of genes in two independent tests. Firstly, we identified genes expressed in female gametocytes that differed significantly in expression compared to asexual stages, as the developmental precursors. This analysis was then repeated for males compared to asexual stages.

In females, we identified 877 genes that had significantly over-represented transcripts compared to asexual stages, and 3212 genes that were under-represented (Fig. 2 c). In males, 1331 genes were over-represented, and 2387 were under-represented. Hence, in females, more genes were under-represented than over-represented, by a factor of 3.66; in males, this factor was only 1.79. Hence, compared to the prior asexual stages, many genes are down-regulated, and comparatively few genes are upregulated, with this phenomenon more striking in females.

We quantified the number of genes common to both types of gametocytes, visualised using a Venn diagram (Fig. 2 c). The greatest subset by far were those genes downregulated in both female and male gametocytes (2110 genes) compared to asexual stages. As an example of this magnitude, 88.4% of downregulated male gametocyte genes were also downregulated in female gametocytes.

We represented these values proportionally on a schematic that depicts the developmentally-ancestral asexual stage differentiating into either male or female gametocytes (Fig. 2 d). In this diagram, the direction of the thick arrows represents either upregulation or downregulation. In addition, the number of differentially expressed genes are indicated on each arrow, with the width of the arrow proportional to this number. After accounting for genes down- or upregulated in both female and male gametocytes (grey arrows), we can identify the remaining genes that are specifically up- or downregulated in female or male gametocytes alone, compared to asexual parasites. In female gametocytes, there are almost three times as many genes downregulated than upregulated (red arrow pair), whereas in males, this is reversed, with three times as many genes being *upregulated* than downregulated (green arrow pair). A similar trend is also observed if we compare the genders directly without reference to asexual stages (green-red arrow pair), with many more genes upregulated in male than female gametocytes. The full list of significantly differentially-expressed genes in provided in Additional files 2 and 3.

We counted the normalised number of transcripts that mapped to each chromosome, and observed over- and under-representation of female and male transcripts on specific chromosomes (Fig. 2 e). This includes a startling over-representation of female transcripts from chromosome five, and an under-representation of female transcripts from chromosome two. On chromosome five, the first and fourth most highly-expressed individual female-specific transcripts compared to asexual stages are the adjacent genes P28 (28 kDa ookinete surface protein; PBANKA_0514900) and P25 (25 kDa ookinete surface antigen precursor; PBANKA_0515000) respectively. These transcripts are over-expressed over 400-fold compared to asexual stages. When reads that mapped to either of these two loci were removed from the analysis, there were no longer any significant differences detected in female transcripts mapping to chromosome five.

### Pathway enrichment analysis

To distill important characteristics from the thousands of differentially-expressed genes, we analysed individual transcriptomes for pathway enrichment. We then removed redundant pathways, and split pathways into biological processes, cellular components, and molecular functions. This left 7 female-specific and 12 male-specific biological processes that are enriched compared to asexual stages; these are presented in accompanying tables (Table 1), with the full lists presented in Additional file 4. Pathways that described cellular components and molecular functions were consistent with the information from biological processes, such as increased cytoskeleton and protein kinase activity in female gametocytes, and increased replication components and DNA binding in male gametocytes. These full lists are also shown in Additional file 4.

**Table 1:** Overexpressed biological processes in female or male gametocytes compared to asexual stages

Gender	GO term ID	GO term description	*p* value	Genes/total
Female-specific	GO:0044403	Symbiosis, encompassing mutualism through parasitism	0.0073	13/31
	GO:0044419	Interspecies interaction between organisms	0.0073	13/31
	GO:0051704	Multi-organism process	0.0073	13/31
	GO:0051701	Interaction with host	0.0086	11/24
	GO:0030260	Entry into host cell	0.0097	10/21
	GO:0006468	Protein phosphorylation	0.013	23/80
	GO:0022402	Cell cycle process	0.046	8/18
Male-specific	GO:0007010	Cytoskeleton organization	4.6 ×10^−17^	37/48
	GO:0006260	DNA replication	2.1 ×10^−16^	35/45
	GO:0007017	Microtubule-based process	5.0 ×10^−15^	30/37
	GO:0006468	Protein phosphorylation	3.8 ×10^−6^	38/80
	GO:0006796	Phosphate-containing compound metabolic process	6.0 ×10^−6^	51/121
	GO:0007018	Microtubule-based movement	8.7 ×10^−6^	15/19
	GO:0030705	Cytoskeleton-dependent intracellular transport	8.7 ×10^−6^	15/19
	GO:0009719	Response to endogenous stimulus	0.0046	22/49
	GO:0022402	Cell cycle process	0.030	10/18
	GO:0006928	Movement of cell or subcellular component	0.031	6/8
	GO:0051674	Localization of cell	0.031	6/8
	GO:0007049	Cell cycle	0.031	12/24

We attempted to identify shared pathways involved in general gametocyte development, by identifying genes simultaneously upregulated in both female and male gametocytes, then analysing for pathway enrichment as above. This strategy only identified one biological process, protein phosphorylation (GO:0006468, *p* value = 0.039, 13/80 genes). We also identified cellular components and molecular functions from this set (see Additional file 4 for full list).

Protein phosphorylation was identified as upregulated in females, males, and shared genes independently. Hence, we further identified the protein kinases involved. We identified upregulation of 23 protein kinases in female gametocytes and 37 protein kinases in male gametocytes, with 13 being shared (see Additional file 5).

Finally, we identified biological processes for genes downregulated in both female and male gametocytes compared to asexual stages (Table 2). Again, cellular components and molecular functions were consistent with these results (see Additional file 4).

**Table 2:** Overexpressed biological processes in asexual stages compared to genes shared by both gametocytes

GO term ID	GO term description	*p* value	Genes/total
GO:0010467	Gene expression	2.1 ×10^−16^	304/436
GO:0006412	Translation	1.0 ×10^−12^	165/217
GO:0009058	Biosynthetic process	1.1 ×10^−8^	236/358
GO:0016070	RNA metabolic process	5.7 ×10^−7^	185/278
GO:0044238	Primary metabolic process	1.5 ×10^−5^	598/1068
GO:0022613	Ribonucleoprotein complex biogenesis	3.36 ×10^−5^	58/73
GO:0043170	Macromolecule metabolic process	5.2 ×10^−5^	533/948
GO:0030163	Protein catabolic process	0.00081	41/51
GO:0009451	RNA modification	0.00095	29/33
GO:0006082	Organic acid metabolic process	0.0042	56/78
GO:0006520	Cellular amino acid metabolic process	0.0046	43/57
GO:0009308	Amine metabolic process	0.012	45/62
GO:0032940	Secretion by cell	0.015	24/29
GO:0046903	Secretion	0.015	24/29
GO:0009057	Macromolecule catabolic process	0.021	50/72
GO:0043038	Amino acid activation	0.022	28/36
GO:0009987	Cellular process	0.033	703/1331
GO:0016192	Vesicle-mediated transport	0.038	42/60
GO:0001522	Pseudouridine synthesis	0.044	15/17
GO:0032259	Methylation	0.044	15/17

### Comparison to known gametocyte-transcript regulation

**Fig. 3:**
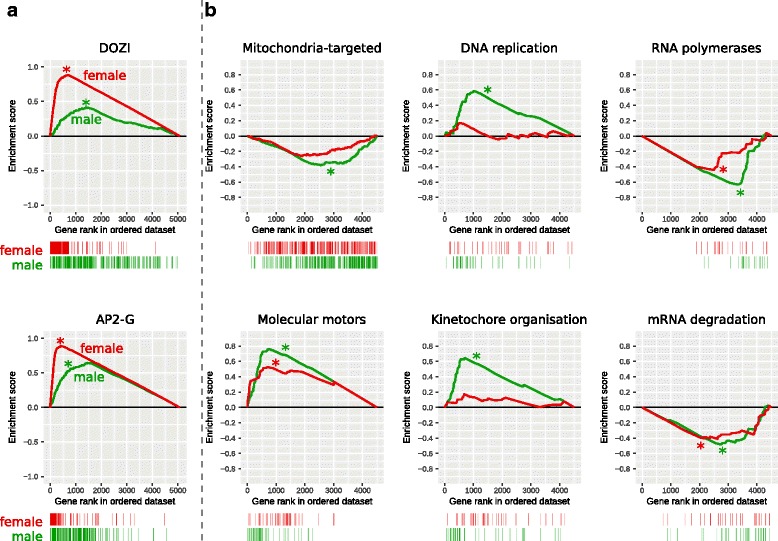
Gene set enrichment analysis. The gametocyte-specific genes are to the left of the x-axis, and the asexual-specific genes are to the right. (See main text for a more extensive explanation). Female enrichment score is plotted with a red line, and male enrichment score with a green line. Statistically significant differences (*p* value < 0.05) are indicated by an asterisk (*). Lines in the “barcode” beneath each plot indicate the position of a gene from that gene set within the ranked list. **a** Enrichment analysis of two *P. berghei* datasets. **b** *P. berghei* genes were transformed into their *P. falciparum*syntenic orthologues, then analysed for enrichment of *P. falciparum* datasets

Previous analyses have identified the RNA binding protein DOZI as an important post-transcriptional regulator required for zygote production [6]. DOZI stabilises and translationally represses transcripts in female gametocytes until their products are required [6]. To test whether genes regulated by DOZI-inactivation had gender-specific transcription, we analysed these for gene set enrichment. We obtained the list of DOZI-regulated genes [6], then used GSEA [16] to determine whether these genes were preferentially transcribed in female and/or male gametocytes. Briefly, genes were ranked based on expression in female stages compared to asexual stages, then arranged in order on the x-axis of a graph (Fig. 3). Female-specific genes are to the left of the graph, and asexual-specific genes are to the right. If a gene in this list is a DOZI-regulated gene, a concomitant vertical stripe is placed under the graph (Fig. 3 a, top, red “barcode”). Hence, if this gene set is female-gametocytes biased, we will see more lines on the left of the barcode; if primarily in asexual stages, to the right of the barcode. We can also quantify the magnitude of this bias with the enrichment score (y-axis). The enrichment score starts at zero for the first gene in the list. As we progress through the list of genes, the presence of a DOZI-regulated gene evokes an increase in the y value, otherwise, the y value drops. The magnitude of these increments is normalised, so that the final gene ends on a zero enrichment score. Hence, if DOZI-regulated genes are primarily female-specific, there will be relatively more early increases than decreases in the enrichment score, and there will be a positive peak. If DOZI-regulated genes are primarily asexual-specific, then there will be a net decrease early on, resulting in a negative enrichment-score peak.

Hence, in female gametocytes, there is a statistically-significant enrichment of DOZI-regulated genes, based on *p* value as estimated using GSEA [16]. A significant enrichment is also observed in male gametocytes, albeit to a lesser degree.

We also tested for gene set enrichment for genes regulated by the gametocyte transcription factors AP2-G or AP2-G2 [3]. We observed upregulation of AP2-G–regulated genes in both female and male gametocytes (Fig. 3 a). We also observed upregulation of AP2-G2 in both gametocyte genders (see Additional file 6). Confusingly, there was a small but significant enrichment of genes in both genders that were also relatively *upregulated* in AP2-G knockouts, and similarly with AP2-G2 in male gametocytes (see Additional file 6). We also assayed for the presence of genes identified as having AP2-G and AP2-G2 motifs [3], but could not establish enrichment of either motif (see Additional file 6).

### Metabolic pathways

We obtained 260 gene sets from Malaria Parasite Metabolic Pathways [17], and performed gene set enrichment analysis for the 187 sets that contained at least 10 genes. We tested female and male gametocyte transcriptomes for enrichment of each of these sets. There were enrichment of 4 sets in female and 23 sets in male gametocytes, compared to asexual stages. Conversely, we observed depletion of 36 and 68 sets in female and male gametocytes respectively.

The full list is presented in Additional file 6. However, we present six biologically-relevant sets in Fig. 3 b. Nuclear-encoded mitochondrially-targeted genes were depleted in both female and male gametocytes, although only significantly so in the latter. Enrichment of molecular motors was exhibited in both genders. Both DNA replication and kinetochore organisation were enriched in male gametocytes but not female gametocytes, congruent with our existing knowledge of the biological processes in these forms. Finally, RNA polymerases and mRNA degradation were depleted in both gametocytes. This is consistent with stabilisation of transcripts known to occur in *Plasmodium* gametocytes [6].

### Motifs

We attempted to identify female- or male-specific sequence motifs by a variety of methods. Firstly, we partitioned transcripts by absolute expression, comparing the top quartile to the bottom quartile. Four separate groups were partitioned: asexual, female-specific, male-specific, and the intersection of female- and male-specific transcripts. The latter would plausibly identify general gametocyte motifs non-specific to either gender. We also repeated this analysis using the top decile instead. We searched for enriched motifs using DREME [18], examining sequences from 1000 kb upstream of the start codon, as per previous analyses in *Plasmodium* [19, 20]. In all cases, we found no convincing gametocyte-specific motif absent in the asexual stages (see Additional file 7).

We then partitioned transcripts by *relative* expression, comparing the most–female-specific transcripts to the most–asexual-specific transcripts. We repeated this for male gametocytes, and then for the intersection of both genders. We were able to identify four motifs conserved in all three groups: the sequence GTCT and its reverse-complement AGAC, and the partial reverse-complements TGTG and CACAC (see Additional file 7 for analyses and sequence logos). However, we were unable to detect any gender-specific motif for either female or male gametocytes.

### Orthology with *P. falciparum*

As previously mentioned, the recent transcriptomic study of male and female gametocytes from *P. falciparum* did not include the asexual developmental precursor as a reference point [13]. Hence, comparisons were limited to direct contrasts of female and male gametocyte, rather than of gametocyte-specific genes. To compare the two species, we restricted our analysis to a similar direct comparison.

Initially, we directly compared sets up- or down-regulated in both studies, using *P. falciparum* syntenic orthologues of our *P. berghei* genes. Focusing on *P. falciparum* female-specific genes, there was moderate overlap, with 39.6% also being female-specific in *P. berghei*. However, there was also considerable disagreement, with 35.6% of these *P. falciparum* female-specific genes overlapping with male-specific genes in *P. berghei* (see Additional file 8).

As mentioned above, the previous study’s results present a useful survey of genes transcribed in *P. falciparum*gametocytes, but this experiment was not designed for statistical analysis of differential expression between genders, or between gametocytes and asexual stages. Therefore, to identify *P. falciparum* and *P. berghei* genes most likely to be meaningfully differentially expressed between male and female gametocytes, we combined a number of analyses based on orthology between the species.

**Fig. 4:**
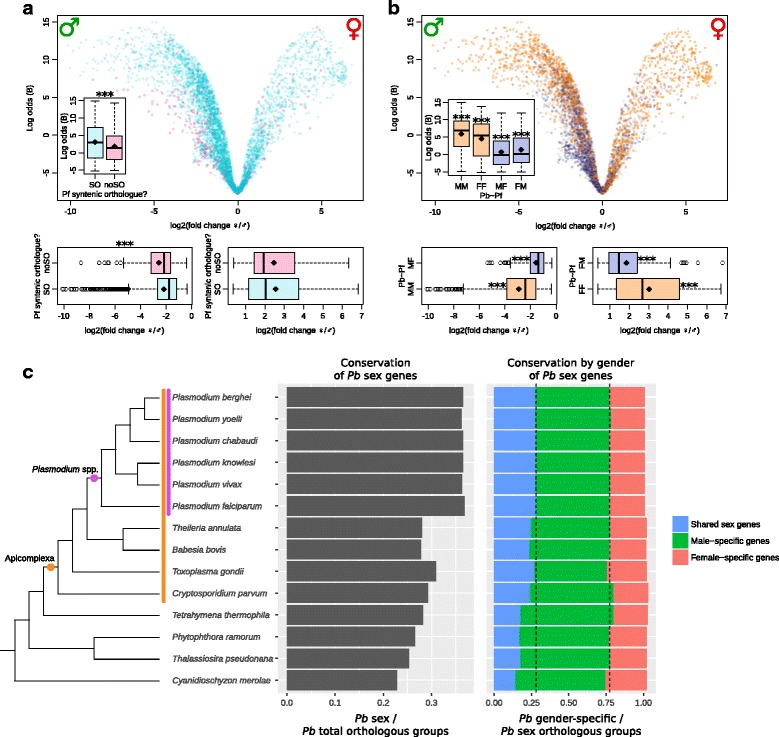
Phylogenetic comparisons of differentially-expressed transcripts. (a and b) Volcano plots of female- and male-specific genes. Each point represents a *P. berghei* gene from our analysis. The y-axis indicates confidence in log odds (B), and the x-axis shows magnitude of difference as log_2_ (fold change ♀/♂), from most male-specific (♂) to most female-specific (♀). Diamond symbols within boxplots indicate the mean. All plots marked *** have *p* value < 0.001 **a** *P. berghei* genes with syntenic orthologues from *P. falciparum* are plotted in cyan; genes lacking syntenic orthologues are shown in pink. Inset boxplots show genes with syntenic orthologues are more likely to have been identified as differentially expressed between genders, based on log odds. Boxplots below the volcano plot demonstrate that genes with syntenic orthologues are more likely to have a lower magnitude of change in male-specific genes, with no difference in female-specific genes. **b** Genes concordant in both species, i.e. male-specific (MM) or female-specific (FF) in both, are marked in orange. Genes that are discordant, i.e. male-specific in *P. berghei* and female-specific in *P. falciparum* (MF) or the opposite (FM), are shown in purple. Inset boxplots show concordant genes are more likely to have been identified in our differential expression screen (higher log odds), and discordant gene are less likely. Boxplots below the volcano plot show that concordant genes have larger magnitude of differential expression, and discordant genes have a lower magnitude. **c** Phylogeny showing conservation of orthologous groups in different organisms. The central panel depicts the number of conserved general sex orthologous groups divided by total orthologous groups for each species. The right panel partitions these sex-related groups into shared sex genes (in both male and female gametocytes), male-specific genes, and female-specific genes. These are normalised for the total number of general-sex orthologous groups. N.B. these need not total 1.00, because a few orthologous groups contain genes from multiple sets. The vertical dashed lines reference the *P. berghei* data on the first row of the phylogeny, and are collinear with the category boundaries for this organism

Firstly, we visualised our results on a volcano plot, which plots statistical confidence on the y-axis (as log odds) and magnitude of change on the x-axis (Fig. 4 a). Hence, points on the left of the graph represent genes highly-expressed in male gametocytes; points on the right are highly-expressed in female gametocytes. Points near the zero point of the x-axis are equivalently expressed in both genders, and are hence associated with a low log odds value. Initially, we determined which genes in our *P. berghei* dataset had a syntenic orthologue in *P. falciparum*. These points are plotted in cyan on the graph; genes without syntenic orthologues are shown in pink. 6.4% of all genes analysed lacked *P. falciparum* syntenic orthologues (304 of 4736 genes). The genes lacking syntenic orthologues were enriched in pathways associated with host cell parts, extraorganismal space, and vacuoles (see Additional file 4 for full list).

We then asked whether *P berghei* genes that possessed *P. falciparum* syntenic orthologues were more or less likely to have been identified in our differential expression screen. We found that genes with syntenic orthologues were more likely to have been identified as differentially expressed in different gametocyte genders, on the basis of log odds (Fig. 4 a, inset). For genes that were male-specific, genes with syntenic orthologues were more likely to have a smaller magnitude of change than those without syntenic orthologues (Fig. 4 a, bottom left). We found no difference in magnitude for genes that were female-specific (Fig. 4 a, bottom right). Many of the lineage-specific genes are encoded by a small number of families, each with many members of variant antigens found on the surface of infected red blood cells [21], so it is unclear how representative these changes are of general gametocyte biology.

Based on the results of the previous analysis, genes without syntenic orthologues were excluded for the subsequent analysis. We mapped syntenic genes from the *P. falciparum* transcriptomic survey [13] onto the remaining *P. berghei* genes (Fig. 4 b). Genes concordant in both studies, i.e. female-specific (FF) or male-specific (MM) in both, are plotted in orange. Genes in disaccord, i.e. male-specific in *P. berghei* and female-specific in *P. falciparum* (MF) or vice versa (FM) are shown in purple. Genes unreported in the *P. falciparum* survey are plotted in grey. This volcano plot displays a clear pattern such that genes in our analysis with a higher fold change between genders, and higher statistical confidence (log odds) are more likely to possess a *P. falciparum*orthologue with the same gender-specific expression.

We then compared each of the first four subsets of genes (MM, FF, MF, FM) independently to the remaining genes. We found that concordant genes were more likely to be identified in our screen in the first place, based on log odds value, whereas discordant genes were not (Fig. 4 b, inset). Similar to the pattern apparent in the volcano plot, concordant genes were statistically more likely to have a greater magnitude of differential expression between gametocyte genders (MM, FF, Fig. 4 b, bottom left and right), whereas discordant genes were more likely to have a lower magnitude of differential expression (MF, FM, Fig. 4 b, bottom left and right). This combination analysis allows reliable identification of shared genes important for sexual differentiation in both *P. berghei* and *P. falciparum*, and also highlights important differences between the two species.

### Broader phylogenetic conservation

In addition to the analysis of *P. falciparum* and *P. berghei* orthologue pairs, we performed a broader phylogenetic analysis, to determine which sex-related genes were conserved in more-distant relatives. We again used the two lists of differential expression data, which compared females or males to asexual stages. General *P. berghei* sex genes were derived from the union of both of these sets. Orthologues of each gene in this list were then identified for a range of organisms, and the presence of genes for each orthologous group tallied for each organism. To normalise the number of sex-related genes, we then repeated this using the entire *P. berghei* genome. For each organism, the number of conserved sex-related orthologous groups were divided by the number of total orthologous groups (Fig. 4 c, centre panel). The normalised number of genes orthologous to *P. berghei* sex-related genes was fairly steady within the *Plasmodium* genus, but dropped abruptly outside this genus.

The *P. berghei* male and female differential expression data were then analysed for overlap; genes were partitioned into female-specific genes, male-specific genes, and shared sex genes (gene upregulated in both genders). Again, all species were analysed for the presence of orthologues. Counts were normalised by dividing by the total number of sex-related orthologous groups in each species (Fig. 4 c, right panel). The relative proportions of these three sets stayed fairly constant within the *Plasmodium* genus. However, the number of shared sex orthologous groups gradually decreased outside this genus, coincident with an increase in male-specific orthologous groups. Female-specific orthologous groups remained fairly constant for all species.

## Discussion

### Differential expression analysis

In this study, we present the complete transcriptomes of *P. berghei* female and male gametocytes. This is the second sequenced transcriptome of separate gametocyte stages of a *Plasmodium* spp. However, it is the first that compares the gametocytes to the asexual developmental-precursor stages, and the first that analyses differential expression of gametocytes based on biological replicates.

We identified 4627 genes differentially expressed in either female or male gametocytes (Fig. 2 c), which represents 91.3% of the 5067 genes analysed. The overall trend was towards downregulation of transcripts in both female and male gametocytes, with the vast majority of these genes downregulated in both genders (Fig. 2 d, black arrow). There was more upregulation of male-specific than female-specific transcripts, and more downregulation of female-specific than male-specific transcripts.

These results are consistent with *P. berghei* proteomic data, with a larger number of male-only proteins absent in asexual stages (236 proteins) compared with female-only proteins absent in asexual stages (101 proteins) [8]. We also observed a large number of transcripts (476 genes; 54% of total female transcripts) that were upregulated in both genders (Fig. 2 c, grey intersection). This contrasts with proteomic data, where a mere 69 proteins (41% of total female proteins) were observed in both genders and absent in asexual stages [8]. This discrepancy may arise from analytical differences due to the higher sensitivity and possibility for replication in high-throughput transcriptomics, differences in female- and male-specific promoters used, and genuine differences between the transcriptome and proteome of gametocytes. Indeed, translational repression is a known mechanism in female gametocytes [6], which is discussed below.

In the *P. falciparum* proteome, from the study that excluded proteins present in the asexual trophozoite stage, 91 male-specific and 171 female-specific proteins were identified, with 349 proteins shared in the two genders [12]. Although the large shared proportion is consistent with our *P. berghei* data, we observed the opposite gender bias, with more male- than female-specific transcripts. This difference would be further exacerbated by transcription repression in female gametocytes, which would imply an ever greater number of female transcripts in *P. falciparum* than the number of proteins suggests. As stated previously, the *P. falciparum* sex-specific transcriptome did not sequence asexual stages, so a statistically-based differential-expression analysis was not previously possible, although the authors observed 78 female-specific transcripts and 10 male-specific transcripts [13].

We observed an over-representation of female transcripts from chromosome 5 (Fig. 2 e). Previously, a cluster of seven genes had been identified on this chromosome (PBANKA_0507400 to PBANKA_0508000), some of which were overexpressed in gametocytes, and were overlapping and/or alternatively spliced [22]. However, we observed no upregulation in female gametocytes for any of these genes, although moderate upregulation was observed in male gametocytes for PBANKA_0507700 to PBANKA_0507900, the latter being increased 32-fold.

Regardless, we identified a novel cluster of female-specific genes on chromosome 5, including P28 and P25, which were very highly expressed. Numerous other surrounding genes also exhibited increased expression compared to asexual stages. This cluster included genes from PBANKA_05148000 to PBANKA_0515400, with seven of the eight genes being female-specific.

### Pathway enrichment analysis

We identified a single biological function upregulated in both gametocytes, namely phosphorylation. This is consistent with our existing knowledge regarding the numerous protein kinases involved in life-cycle progression just after gametocytogenesis [23, 24]. This function was also enriched in female and male gametocytes independently.

Female-specific protein kinases that were upregulated include NEK4, which had been previously identified in the female gametocyte proteome [8]. NEK4 is an essential protein kinase in female gametocytes for development into the subsequent ookinete stage [25]. Male-specific protein kinases identified include MAPK2 and CDPK4; peptides of the former had been previously identified in male gametocytes, although the latter had been identified in both genders [8]. Both of these protein kinases are essential for male gametocyte exflagellation [26–28]. Finally, transcripts for several protein kinases were upregulated in both genders, including CDPK3 and NEK2, neither of which had been identified in the *P. berghei* shared-gender gametocyte proteome [8]. These protein kinases are involved in ookinete motility and development [29–31]. In total, we identified 47 protein kinases upregulated in either gametocyte gender, including 24 that had not been previously investigated (see supplementary data A for full list). These are promising candidates for further investigation of the differential regulation of male and female gametocytes, and include potential female-specific protein kinases, including a putative serine/threonine protein kinase (PBANKA_1305200) and the protein kinase PBANKA_1414500. The former is upregulated 170-fold in female gametocytes, and only 4.7-fold in male gametocytes; the latter is upregulated 310-fold in females and 17-fold in males. This list includes male-specific protein kinases, such as the serine/threonine protein kinase PK9 (PBANKA_1413600), which is unchanged in females, but upregulated 37-fold in males. This gene has been relatively unstudied, but is known to target a ubiquitin-conjugating enzyme [32].

In female gametocytes, the vast majority of biological processes relate to the interaction with hosts (Table 1). This may correspond with later fusion with male gametes, or ookinete penetration of the mosquito midgut.

In male gametocytes, we identified a swathe of cytoskeletal, microtubule and movement-related biological processes, which are transcribed in preparation for the motile male gamete stage that follows. This is consistent with the subsequent presence of flagellar proteins in the proteome of *P. berghei* male gametes [33]; however, we were unable to detect any significant increase of transcripts relating to glycolytic pathways, despite these proteins being observed in male gametes [33]. We also found upregulated pathways involved in DNA replication and related processes. These are presumably necessary for the rapid rounds of DNA replication that occur just before male gametocyte exflagellation into gametes [10]. We also found an enrichment of pathways involved in responses to endogenous stimuli, which is consistent with the sensing of mosquito-environment triggers that induce exflagellation, such as xanthurenic acid [9].

### Comparison to known gametocyte-transcript regulation

We found a very strong correlation between DOZI-regulated genes and highly-expressed female transcripts. We also observed a strong, albeit lesser, correlation with male transcripts (Fig. 3 a). This was consistent with male gametocytes producing less DOZI than female gametocytes [8], and female DOZI null mutants being fully sterile, compared to male DOZI null mutants, which are unaffected in fertility [6].

There was a strong correlation between genes induced by gametocyte regulators AP2-G and AP2-G2 with high expression in both gametocytes (Fig. 3 a). Surprisingly, we could detect no enrichment of genes previously identified as having a motif associated with these regulators [3], which may suggest that the regulators directly affect only a small number of genes.

### Metabolic pathways

We observed a significant depletion of mitochondrially-targeted genes in male gametocytes, and a non-significant reduction in females. This is consistent with the presence of more mitochondrial peptides in female gametocytes than males [8], although our observed depletion in gametocytes compared to asexual stages is the opposite of other (non–gender-specific) proteomic and microarray data [34]. However, an upregulation of mitochrondrial transcripts in female gametocytes relative to asexual stages is consistent with the previously-reported upregulation of proteins in the ookinete stage [34]. The depletion of transcripts for mitochondrial proteins in male gametocytes, and the slight but non-significant depletion in females is *prima facie* at odds with previous observations of upregulated mitochondrial metabolism [35], enlarged and elaborate mitochondria [36], and the more elaborate tubular cristae of gametocyte mitochondria [37]. However, despite an increased metabolic output, no organellar division is apparent in gametocyte stages [36], and male gametes appear to lack mitochondria [5, 36]. Hence, the gametocyte mitochondria may require a lower turnover of proteins and thus lower transcription of genes for mitochondrial-targeted proteins.

We also saw a concomitant reduction of apicoplast-targeted genes in male gametocytes, and a non-significant depletion in female stages (see Additional file 6). Similarly to mitochondria, apicoplasts are only maternally inherited [38, 39] and male gametes appear to be entirely lacking in apicoplasts [36].

We found an enrichment of molecular motors in both genders, but particularly in male gametocytes, which is consistent with the centrality of motility to male gamete function. After fertilisation of gametes, the zygote develops into a motile ookinete, which may explain the presence of motor transcripts in female gametocytes.

DNA replication and chromosome separation (via kinetochore organisation) were enriched only in male gametocytes, which is again consistent with the rapid rounds of DNA replication immediately prior to male gametocyte exflagellation [10].

General RNA processing appears to be reduced, with depletion of both RNA polymerases and mRNA degradation in both genders. This implies a relatively static pool of mRNA, a large proportion of which is presumably held in the translationally-inactive DOZI- or CITH-bound pool. Although male gametocytes have a weaker relationship with DOZI, they have a low amount of translation in general [5], which is consistent with reduced RNA processing.

### Motifs

As mentioned previously, we were unable to find a correlation between highly-expressed female- or male-gametocyte genes with genes previously identified by others as possessing motifs responsible for binding the gametocyte transcription factors AP2-G or AP2-G2 [3]. Consistent with this, our own de-novo motif-discovery analysis did not identify any of these motifs either (GxGTAC or GTACxC for AP2-G, and TGCxACC or GGTxGCA for AP2-G2 [3]). This may imply that those AP2 transcription factors are responsible for initiating a cascade of gametocyte genes, but may not themselves serve as the direct transcription factors for most gametocyte-expressed genes.

We found no motifs specific to either gender, but did find motifs conserved in both gametocyte genders and the intersection of both. These were the sequence GTCT and its reverse-complement AGAC, and the partial reverse-complements TGTG and CACAC. We compared these motifs to known ApiAP2 regulatory motifs [40]. The first pair had not been previously identified, but CACACA had been reported as a gametocyte-specific motif [40].

### Orthology with *P. falciparum*

*P. berghei* genes without syntenic orthologues in *P. falciparum*, of which a large proportion are lineage-specific genes, were less likely to be detected as female- or male-specific in our study. Many of these belong to large families of red-blood-cell remodelling and virulence factors and are unlikely to play a gender-specific role in gametocyte biology [21]. For males, these lineage-specific genes were also more likely to have a smaller difference in expression compared to asexual stages. These results are counter-intuitive, because log odds are generally associated with a larger magnitude of differential expression. Genes identified as male- or female-specific in a previous survey of *P. falciparum* transcripts showed strong correlation with our data, although as previously stated, the difference in experimental designs prevented an exact comparison of differentially expressed genes.

### Broader phylogenetic conservation

*P. berghei* sex-related genes are strongly conserved in the *Plasmodium* genus, but contrary to our expectation that sex-related genes would be highly conserved among related eukaryotes that undergo sex, these genes are less conserved than non–sex-related genes in other eukaryotes (Fig. 4 c, centre panel). This suggests that the molecules responsible for *P. berghei* sex are relatively specific to this genus. By extension, given this conservation, the sex machinery in all *Plasmodium* species is highly-specific to this genus. An alternative explanation is that these genes are not sex-specific *per se*, but are genes necessary for gametocytes preparing for their mosquito host, a life strategy not shared by other eukaryotes in our analysis.

In this context, it is noteworthy that some close relatives of *Plasmodium* also parasitise erythrocytes (e.g. *Babesia*, *Theileria*, Haemoproteidae, *Leucocytozoon*), but most do not infect mosquitoes. Some do not even infect insects, so a mosquito life-strategy is presumably a derived character in *Plasmodium.* However, the explanation that these poorly-conserved genes are merely mosquito-adapted genes is not strongly supported by an analysis of the gender-specific gametocyte genes. Such a hypothesis would predict that conservation of generic gametocyte genes would decay in alveolates lacking insect hosts, while gender-specific genes remained relatively well-conserved. This is not borne out by our analysis; instead the proportions of male, female and shared gametocytes show no dramatic change between the lineages with and without dipteran or other insect hosts (Fig. 4 c).

Nonetheless, there are some changes in relative conservation of gender-specific genes. While the female-specific proportion of sex genes were conserved fairly evenly through the organisms analysed, there was a clear decline in conservation of the genes shared between gametocytes, and a concomitant increase in the male-specific proportion. This appears in part to represent a broad conservation of male-enriched sex genes, such as flagellation, DNA replication and cytokinesis (see Table 1), all of which are typical characteristics of maleness in the Alveolata, i.e. being smaller, more motile, and more numerous. Female-enriched genes are moderately conserved, whereas shared-sex genes are poorly conserved, particularly outside the Apicomplexa. Hence, as this genus diverged, it co-opted the existing sexual mechanisms for sex that it could, but also required novel sex genes. These were sometimes shared in both genders, a frugal evolution strategy, consistent with the compact genome of *Plasmodium*.

## Conclusions

We have performed the first transcriptomic analysis of separated female and male gametocytes in *P. berghei*, and the first differential-expression analysis in any *Plasmodium* species. We have identified broad tendencies towards net downregulation of genes in gametocytes compared to asexual stages, with this more prominent in females. Confirmation of pathways consistent with current knowledge of gametocyte biology has validated our analysis. We have additionally identified numerous new female- and male-specific pathways and genes for further analysis, including several novel gender-specific protein kinases.

We determined the extent of evolutionary conservation of sex-related genes between *P. berghei* and related organisms. Sex-related genes were highly-conserved in the *Plasmodium* genus, and less so in other eukaryotes. Genes shared by both genders exhibited a similar distribution, in contrast to male-specific genes, which showed better conservation outside this genus. These data suggest that many genes involved in *Plasmodium* sex evolved within this genus.

## Methods

### Experimental animals and parasites

Experiments were performed in conformity with the Australian Prevention of Cruelty to Animals Act 1986 and the Prevention of Cruelty to Animals Regulations 2008, and reviewed and permitted by the Melbourne University Animal Ethics Committee (AEC ethics ID: 1413078). Male Swiss Webster mice aged four to six weeks old were used in all experiments. These mice were purchased from the Monash Animal Research Platform, Melbourne.

We obtained transgenic *P. berghei* ANKA 820cl1m1cl1 parasites (a kind gift from Andy Waters, University of Glasgow, Glasgow, Scotland) [14], which were subsequently passed through the entire life cycle, to ensure healthy gametocytes. All parasites sequenced had been cultured in one to three sequential mice after mosquito infection.

### Purification of male and female gametocytes

“Pre-infection” mice were infected with thawed parasites by intraperitoneal injection, with parasitemia monitored by Giemsa-stained smears. One day later, naive “experimental” mice were injected with 200 *μ*l phenylhydrazine (6 mg/ml). Three to six days after this, parasites were obtained from “pre-infection” mice by cardiac bleed. “Experimental” mice were immediately infected by intraperitoneal injection of 200 *μ*l of fresh parasites from this stock.

Three days later, “experimental” mice were humanely killed with 20% slow-fill carbon dioxide in accordance with the ethics approval and regulations above. This was immediately followed by direct cardiac puncture. Parasites were immediately diluted into 4° C DPBS (Dulbecco’s phosphate-buffered saline), and kept on 4° C cold packs. Samples were enriched for gametocytes, trophozoites, and schizonts via differential centrifugation in a Nycodenz ^*Ⓡ*^ (Sigma-Aldrich, Australia) solution, using a slightly-modified protocol to that described by Janse and colleagues [41]. Briefly, a 49% (v/v) Nycodenz ^*Ⓡ*^ -DPBS solution was used, which equates to a final concentration of 13.5% (w/v) Nycodenz ^*Ⓡ*^. 4° C solutions and cold packs were used in all stages, and the centrifuge was pre-cooled to 4° C.

Parasites were sorted in a cooled PC2 MoFlo ^*Ⓡ*^ Astrios^TM^ cell sorter. Purified male and female gametocytes were immediately concentrated by centrifugation, then frozen at −80° C.

### Enrichment of asexual erythrocytic stages

Mice were infected with thawed parasites by intraperitoneal injection. Parasitemia was monitored by Giemsa-stained smears. When an appropriate parasitemia was reached, mice were humanely killed and cardiac bled as above. Samples were immediately passed through hand-packed CF11 cellulose columns, to remove leukocytes and platelets [42], then immediately lysed with 0.15% saponin and 0.1% bovine serum albumin in DPBS at 4° C for 10 min to remove erythrocyte transcripts, before washing thrice with DPBS.

### RNA extraction and sequencing

RNA was obtained from frozen purified gametocytes and fresh asexual stages, with an ISOLATE II RNA Mini Kit (Bioline, Australia), as per the manufacturer’s instructions. This was provided to AGRF (Melbourne) for cDNA library construction and mRNA sequencing (by poly-A enrichment) on an Illumina HiSeq2500 (100 bp, strand-specific, paired-end reads).

### Bioinformatic analyses

Analyses were completed on a Lenovo x86 supercomputer (Melbourne Bioinformatics, Australia), an IBM iDataplex x86 supercomputer (Melbourne Bioinformatics, Australia), or personal computers. Reads were mapped and quantified as previously described [43]. Briefly, reads were mapped with Tophat2 [44], visualised in IGV [45], and checked with FastQC [46] and flagstats [47]. Differential-expression and RPKM analyses were performed with limma/voom [48] a package in R [49]. Heatmaps were created from RPKM values using heatmap.2, which is provided by gplots [50] in R.

With the exception of flow cytometry data (Fig. 1 b), all graphs were created in R [49], using the packages beeswarm [51] (Fig. 2 e) or ggplot2 [52] (Figs. 3 and 4). Statistical difference was assessed in R using Welch’s two-sample t-test (Figs. 2 e, 4 a and 4 b). Chromosome-specific counts (Fig. 2 e) were normalised firstly to aggregate read depth for each sample, then to the mean of the three asexual-stage samples for each chromosome; Bonferroni correction for multiple hypothesis testing was also applied. *p* values of <0.05 were considered statistically significant for all tests.

Pathway enrichment was analysed with GOstat [53], based on gene ontology categories extracted from PlasmoDB [54]. Gene ontology categories were collapsed with REVIGO [55], with medium (0.7) similarity and using sizes from *P. falciparum* GO terms.

Gene set enrichment analyses were performed with GSEA [16], running 10 000 permutations, without collapsing dataset to gene symbols, defining permutation type as gene set, reducing the minimum size to 10 members, and removing the maximum size. We used previously-published *P. berghei* lists of genes affected by DOZI [6] and AP2-G/AP2-G2 [3]. Metabolic pathways were obtained using *P. falciparum* data available from Malaria Parasite Metabolic Pathways [17], which were then mapped to syntenic orthologues in *P. berghei*.

Motifs were detected using DREME [18], running discriminative mode, and scanning the given strand only. We analysed sequences from 1000 kb upstream of the start codon, as per previous analyses in *Plasmodium* [19, 20]. Motifs with fewer than four nucleotides were removed.

Comparison with the previously-published *P. falciparum* transcriptome of separated female and male gametocytes was achieved by mapping the published data onto *P. berghei* syntenic orthologues, then processing the output of limma using R [49]. For pairwise statistical comparisons (Fig. 4 a and b), only genes from our limma output with *p* value < 0.05 were included.

The phylogeny (Fig. 4 c) was obtained by merging published *Plasmodium* [56–58] and apicomplexan trees [59, 60]. Orthology of upregulated genes from limma’s output was established using groups specified by OrthoMCL release 5 [61].

All images were created, edited and/or arranged in Inkscape [62]. The manuscript was prepared using LyX [63] and vim [64], with all other text files manipulated with the latter. Detailed commands including version numbers are contained in Additional file 9.

## Additional files

Additional file 1 A tab-delimited text file containing the normalised RPKM counts for transcripts from all samples. (TSV 807 kb)

Additional file 2 A tab-delimited text file containing the full list of genes differentially expressed in female gametocytes compared to asexual erthythrocytic stages. (TSV 674 kb)

Additional file 3 A tab-delimited text file containing the full list of genes differentially expressed in male gametocytes compared to asexual erthythrocytic stages. (TSV 612 kb)

Additional file 4 A zip-compressed file of tab-delimited text files, containing the full list of upregulated pathways in female gametocytes compared to asexual stages, male gametocytes compared to asexual stages, shared gametocyte genes either up- or downregulated compared to asexual stages, and *P. berghei* genes lacking *P. falciparum* orthologues. (ZIP 27 kb)

Additional file 5 A tab-delimited text file comparing differential expression of protein kinases in female and male gametocytes. (TSV 8 kb)

Additional file 6 A zip-compressed file of tab-delimited text files, containing gene set enrichment analyses, comparing either female or male gametocytes to asexual stages. Data include normalised enrichment score (NES), corresponding with the extent of enrichment, and adjusted *p* values for multiple hypothesis testing. (ZIP 8 kb)

Additional file 7 A PDF image containing sequence logos and unerased e-values for all identified motifs four nucleotides and longer. (PDF 166 kb)

Additional file 8 A PDF image comparing the gender-specific transcripts identified in *P. berghei* compared to those previously identified in *P. falciparum*. (PDF 11 kb)

Additional file 9 A text file containing additional bioinformatic methods not in the main article. Includes commands and versioning of software used in this study. (TXT 2 kb)

## Abbreviations

AP2-G: Apetala 2–gametocyte
AP2-G2: Apetala 2–gametocyte 2
CITH: CAR-I/Trailer Hitch homolog
DPBS: Dulbecco’s phosphate-buffered saline
DOZI: Development of zygote inhibited
ER: Endoplasmic reticulum
GFP: Green fluorescent protein
P25: 25 kDa ookinete surface antigen precursor
P28: 28 kDa ookinete surface protein
RFP: Red fluorescent protein
v/v: Volume per volume
w/v: Mass (gram) per volume (litre)

## References

[1] Santiago-Alarcon D, Palinauskas V, Schaefer HM (2012). Diptera vectors of avian Haemosporidian parasites: untangling parasite life cycles and their taxonomy. Biol Rev Camb Philos Soc.

[2] Martinsen ES, Perkins SL, Schall JJ (2008). A three-genome phylogeny of malaria parasites (*Plasmodium* and closely related genera): evolution of life-history traits and host switches. Mol Phylogenet Evol.

[3] Sinha A, Hughes KR, Modrzynska KK, Otto TD, Pfander C, Dickens NJ, Religa AA, Bushell E, Graham AL, Cameron R, Kafsack BFC, Williams AE, Llinás M, Berriman M, Billker O, Waters AP (2014). A cascade of dna-binding proteins for sexual commitment and development in *Plasmodium*. Nature.

[4] Mons B, Janse CJ, Boorsma EG, Van der Kaay HJ (1985). Synchronized erythrocytic schizogony and gametocytogenesis of *Plasmodium berghei* in vivo and in vitro. Parasitology.

[5] Janse CJ, Waters AP, Waters AP, Janse CJ (2004). Sexual development of parasites. Malaria Parasites: Genomes and Molecular Biology.

[6] Mair GR, Braks JAM, Garver LS, Wiegant JCAG, Hall N, Dirks RW, Khan SM, Dimopoulos G, Janse CJ, Waters AP (2006). Regulation of sexual development of *Plasmodium* by translational repression. Science.

[7] Mair GR, Lasonder E, Garver LS, Franke-Fayard BMD, Carret CK, Wiegant JCAG, Dirks RW, Dimopoulos G, Janse CJ, Waters AP (2010). Universal features of post-transcriptional gene regulation are critical for *Plasmodium* zygote development. PLoS Pathog.

[8] Khan SM, Franke-Fayard B, Mair GR, Lasonder E, Janse CJ, Mann M, Waters AP (2005). Proteome analysis of separated male and female gametocytes reveals novel sex-specific *Plasmodium* biology. Cell.

[9] Billker O, Lindo V, Panico M, Etienne AE, Paxton T, Dell A, Rogers M, Sinden RE, Morris HR (1998). Identification of xanthurenic acid as the putative inducer of malaria development in the mosquito. Nature.

[10] Janse CJ, Ponnudurai T, Lensen AH, Meuwissen JH, Ramesar J, Van der Ploeg M, Overdulve JP (1988). DNA synthesis in gametocytes of *Plasmodium falciparum*. Parasitology.

[11] Sinden RE, Alavi YIH, Butcher GA, Dessens JT, Raine JD, Trueman HE, Waters AP, Janse CJ (2004). Ookinete cell biology. Malaria Parasites: Genomes and Molecular Biology.

[12] Miao J, Chen Z, Wang Z, Shrestha S, Li X, Li R, Cui L (2017). Sex-specific biology of the human malaria parasite revealed from the proteomes of mature male and female gametocytes. Mol Cell Proteomics.

[13] Lasonder E, Rijpma SR, van Schaijk BCL, Hoeijmakers WAM, Kensche PR, Gresnigt MS, Italiaander A, Vos MW, Woestenenk R, Bousema T, Mair GR, Khan SM, Janse CJ, Bártfai R, Sauerwein RW (2016). Integrated transcriptomic and proteomic analyses of *P. falciparum* gametocytes: molecular insight into sex-specific processes and translational repression. Nucleic Acids Res.

[14] Ponzi M, Sidén-Kiamos I, Bertuccini L, Currà C, Kroeze H, Camarda G, Pace T, Franke-Fayard B, Laurentino EC, Louis C, Waters AP, Janse CJ, Alano P (2009). Egress of *Plasmodium berghei* gametes from their host erythrocyte is mediated by the MDV-1/PEG3 protein. Cell Microbiol.

[15] Franke-Fayard B, Janse CJ, Cunha-Rodrigues M, Ramesar J, Büscher P, Que I, Löwik C, Voshol PJ, den Boer MAM, van Duinen SG, Febbraio M, Mota MM, Waters AP (2005). Murine malaria parasite sequestration: CD36 is the major receptor, but cerebral pathology is unlinked to sequestration. Proc Natl Acad Sci U S A.

[16] Subramanian A, Tamayo P, Mootha VK, Mukherjee S, Ebert BL, Gillette MA, Paulovich A, Pomeroy SL, Golub TR, Lander ES, Mesirov JP (2005). Gene set enrichment analysis: a knowledge-based approach for interpreting genome-wide expression profiles. Proc Natl Acad Sci U S A.

[17] Ginsburg H, Abdel-Haleem AM (2016). Malaria parasite metabolic pathways (MPMP) upgraded with targeted chemical compounds. Trends Parasitol.

[18] Bailey TL (2011). DREME: motif discovery in transcription factor ChIP-seq data. Bioinformatics.

[19] Harris EY, Ponts N, Le Roch KG, Lonardi S (2011). Chromatin-driven de novo discovery of DNA binding motifs in the human malaria parasite. BMC Genomics.

[20] Jurgelenaite R, Dijkstra TMH, Kocken CHM, Heskes T (2009). Gene regulation in the intraerythrocytic cycle of *Plasmodium falciparum*. Bioinformatics.

[21] Sargeant TJ, Marti M, Caler E, Carlton JM, Simpson K, Speed TP, Cowman AF (2006). Lineage-specific expansion of proteins exported to erythrocytes in malaria parasites. Genome Biol.

[22] van Lin LH, Pace T, Janse CJ, Birago C, Ramesar J, Picci L, Ponzi M, Waters AP (2001). Interspecies conservation of gene order and intron-exon structure in a genomic locus of high gene density and complexity in *Plasmodium*. Nucleic Acids Res.

[23] Tewari R, Straschil U, Bateman A, Böhme U, Cherevach I, Gong P, Pain A, Billker O (2010). The systematic functional analysis of *Plasmodium* protein kinases identifies essential regulators of mosquito transmission. Cell Host Microbe.

[24] Carvalho TG, Morahan B, John von Freyend S, Boeuf P, Grau G, Garcia-Bustos J, Doerig C (2016). The ins and outs of phosphosignalling in *Plasmodium*: Parasite regulation and host cell manipulation. Mol Biochem Parasitol.

[25] Reininger L, Billker O, Tewari R, Mukhopadhyay A, Fennell C, Dorin-Semblat D, Doerig C, Goldring D, Harmse L, Ranford-Cartwright L, Packer J, Doerig C (2005). A NIMA-related protein kinase is essential for completion of the sexual cycle of malaria parasites. J Biol Chem.

[26] Billker O, Dechamps S, Tewari R, Wenig G, Franke-Fayard B, Brinkmann V (2004). Calcium and a calcium-dependent protein kinase regulate gamete formation and mosquito transmission in a malaria parasite. Cell.

[27] Rangarajan R, Bei AK, Jethwaney D, Maldonado P, Dorin D, Sultan AA, Doerig C (2005). A mitogen-activated protein kinase regulates male gametogenesis and transmission of the malaria parasite *Plasmodium berghei*. EMBO Rep.

[28] Tewari R, Dorin D, Moon R, Doerig C, Billker O (2005). An atypical mitogen-activated protein kinase controls cytokinesis and flagellar motility during male gamete formation in a malaria parasite. Mol Microbiol.

[29] Siden-Kiamos I, Ecker A, Nybäck S, Louis C, Sinden RE, Billker O (2006). *Plasmodium berghei* calcium-dependent protein kinase 3 is required for ookinete gliding motility and mosquito midgut invasion. Mol Microbiol.

[30] Ishino T, Orito Y, Chinzei Y, Yuda M (2006). A calcium-dependent protein kinase regulates *Plasmodium* ookinete access to the midgut epithelial cell. Mol Microbiol.

[31] Reininger L, Tewari R, Fennell C, Holland Z, Goldring D, Ranford-Cartwright L, Billker O, Doerig C (2009). An essential role for the *Plasmodium* Nek-2 Nima-related protein kinase in the sexual development of malaria parasites. J Biol Chem.

[32] Philip N, Haystead TA (2007). Characterization of a UBC13 kinase in *Plasmodium falciparum*. Proc Natl Acad Sci U S A.

[33] Talman AM, Prieto JH, Marques S, Ubaida-Mohien C, Lawniczak M, Wass MN, Xu T, Frank R, Ecker A, Stanway RS, Krishna S, Sternberg MJE, Christophides GK, Graham DR, Dinglasan RR, Yates JR, Sinden RE (2014). Proteomic analysis of the *Plasmodium* male gamete reveals the key role for glycolysis in flagellar motility. Malar J.

[34] Hall N, Karras M, Raine JD, Carlton JM, Kooij TWA, Berriman M, Florens L, Janssen CS, Pain A, Christophides GK, James K, Rutherford K, Harris B, Harris D, Churcher C, Quail MA, Ormond D, Doggett J, Trueman HE, Mendoza J, Bidwell SL, Rajandream MA, Carucci DJ, Yates JR, Kafatos FC, Janse CJ, Barrell B, Turner CMR, Waters AP, Sinden RE (2005). A comprehensive survey of the *Plasmodium* life cycle by genomic, transcriptomic, and proteomic analyses. Science.

[35] Srivastava A, Philip N, Hughes KR, Georgiou K, MacRae JI, Barrett MP, Creek DJ, McConville MJ, Waters AP (2016). Stage-specific changes in *Plasmodium* metabolism required for differentiation and adaptation to different host and vector environments. PLoS Pathog.

[36] Okamoto N, Spurck TP, Goodman CD, McFadden GI (2009). Apicoplast and mitochondrion in gametocytogenesis of *Plasmodium falciparum*. Eukaryot Cell.

[37] Krungkrai J, Prapunwattana P, Krungkrai SR (2000). Ultrastructure and function of mitochondria in gametocytic stage of *Plasmodium falciparum*. Parasite.

[38] Creasey A, Mendis K, Carlton J, Williamson D, Wilson I, Carter R (1994). Maternal inheritance of extrachromosomal DNA in malaria parasites. Mol Biochem Parasitol.

[39] Ferguson DJP, Henriquez FL, Kirisits MJ, Muench SP, Prigge ST, Rice DW, Roberts CW, McLeod RL (2005). Maternal inheritance and stage-specific variation of the apicoplast in *Toxoplasma gondii* during development in the intermediate and definitive host. Eukaryot Cell.

[40] Campbell TL, De Silva EK, Olszewski KL, Elemento O, Llinás M (2010). Identification and genome-wide prediction of DNA binding specificities for the ApiAP2 family of regulators from the malaria parasite. PLoS Pathog.

[41] Janse CJ, Ramesar J, Waters AP (2006). High-efficiency transfection and drug selection of genetically transformed blood stages of the rodent malaria parasite *Plasmodium berghei*. Nat Protoc.

[42] Sriprawat K, Kaewpongsri S, Suwanarusk R, Leimanis ML, Lek-Uthai U, Phyo AP, Snounou G, Russell B, Renia L, Nosten F (2009). Effective and cheap removal of leukocytes and platelets from *Plasmodium vivax* infected blood. Malar J.

[43] Yeoh LM, Goodman CD, Hall NE, van Dooren GG, McFadden GI, Ralph SA (2015). A serine-arginine–rich (SR) splicing factor modulates alternative splicing of over a thousand genes in *Toxoplasma gondii*. Nucleic Acids Res.

[44] Kim D, Pertea G, Trapnell C, Pimentel H, Kelley R, Salzberg SL (2013). TopHat2: accurate alignment of transcriptomes in the presence of insertions, deletions and gene fusions. Genome Biol.

[45] Thorvaldsdóttir H, Robinson JT, Mesirov JP (2013). Integrative Genomics Viewer (IGV): high-performance genomics data visualization and exploration. Briefings Bioinf.

[46] Andrews S. FastQC 0.11.5: A Quality Control Tool for High Throughput Sequence data. 2016. http://www.bioinformatics.babraham.ac.uk/projects/fastqc/. Accessed 14 Feb 2017.

[47] Li H, Handsaker B, Wysoker A, Fennell T, Ruan J, Homer N, Marth G, Abecasis G, Durbin R, GPDPS (2009). The Sequence Alignment/Map format and SAMtools. Bioinformatics.

[48] Law CW, Chen Y, Shi W, Smyth GK (2014). Voom: precision weights unlock linear model analysis tools for RNA-seq read counts. Genome Biol.

[49] R Core Team (2017). R: A Language and Environment for Statistical Computing.

[50] Warnes GR, Bolker B, Bonebakker L, Gentleman R, Liaw WHA, Lumley T, Maechler M, Magnusson A, Moeller S, Schwartz M, Venables B. Gplots: Various R Programming Tools for Plotting Data. 2016. R package version 3.0.1. https://CRAN.R-project.org/package=gplots. Accessed 25 Aug 2017.

[51] Eklund A. Beeswarm: The Bee Swarm Plot, an Alternative to Stripchart. 2016. R package version 0.2.3. https://CRAN.R-project.org/package=beeswarm. Accessed 25 Aug 2017.

[52] Wickham H (2009). Ggplot2: Elegant Graphics for Data Analysis.

[53] Beissbarth T, Speed TP (2004). GOstat: find statistically overrepresented Gene Ontologies within a group of genes. Bioinformatics.

[54] Aurrecoechea C, Brestelli J, Brunk BP, Dommer J, Fischer S, Gajria B, Gao X, Gingle A, Grant G, Harb OS, Heiges M, Innamorato F, Iodice J, Kissinger JC, Kraemer E, Li W, Miller JA, Nayak V, Pennington C, Pinney DF, Roos DS, Ross C, Stoeckert CJJr, Treatman C, Wang H (2009). PlasmoDB: a functional genomic database for malaria parasites. Nucleic Acids Res.

[55] Supek F, Bošnjak M, Škunca N, Šmuc T (2011). REVIGO summarizes and visualizes long lists of gene ontology terms. PLoS One.

[56] Hoo R, Zhu L, Amaladoss A, Mok S, Natalang O, Lapp SA, Hu G, Liew K, Galinski MR, Bozdech Z, Preiser PR (2016). Integrated analysis of the *Plasmodium* species transcriptome. EBioMedicine.

[57] Escalante AA, Freeland DE, Collins WE, Lal AA (1998). The evolution of primate malaria parasites based on the gene encoding cytochrome b from the linear mitochondrial genome. Proc Natl Acad Sci U S A.

[58] Carlton JM, Escalante AA, Neafsey D, Volkman SK (2008). Comparative evolutionary genomics of human malaria parasites. Trends Parasitol.

[59] Arisue N, Hashimoto T (2015). Phylogeny and evolution of apicoplasts and apicomplexan parasites. Parasitol Int.

[60] Kuo CH, Wares JP, Kissinger JC (2008). The apicomplexan whole-genome phylogeny: an analysis of incongruence among gene trees. Mol Biol Evol.

[61] Chen F, Mackey AJ, Stoeckert CJJr, Roos DS (2006). OrthoMCL-DB: querying a comprehensive multi-species collection of ortholog groups. Nucleic Acids Res.

[62] The Inkscape Team. Inkscape 0.92.1. 2017. http://www.inkscape.org. Accessed 28 Mar 2017.

[63] The LyX Team. LyX 2.2.2 — The Document Processor. 2016. http://www.lyx.org/. Accessed 28 Mar 2017.

[64] Moolenaar B, et al.Vim 8.0.0427. 2017. http://www.vim.org/. Accessed 28 Mar 2017.

[65] Leinonen R, Sugawara H, Shumway M, Collaboration INSD (2011). The sequence read archive. Nucleic Acids Res.

[66] Edgar R, Domrachev M, Lash AE (2002). Gene Expression Omnibus: NCBI gene expression and hybridization array data repository. Nucleic Acids Res.

