# Supplementary material for: Comparative transcriptomics of female and male gametocytes in Plasmodium berghei and the evolution of sex in alveolates
Source: BMC Genomics. 2017 Sep 18;18:734. doi: 10.1186/s12864-017-4100-0 (PMC5604118; doi:10.1186/s12864-017-4100-0)
Supplement: Supplementary file 7 — A PDF image containing sequence logos and unerased e-values for all identified motifs four nucleotides and longer. (PDF 166 kb) [file 12864_2017_4100_MOESM7_ESM.pdf]

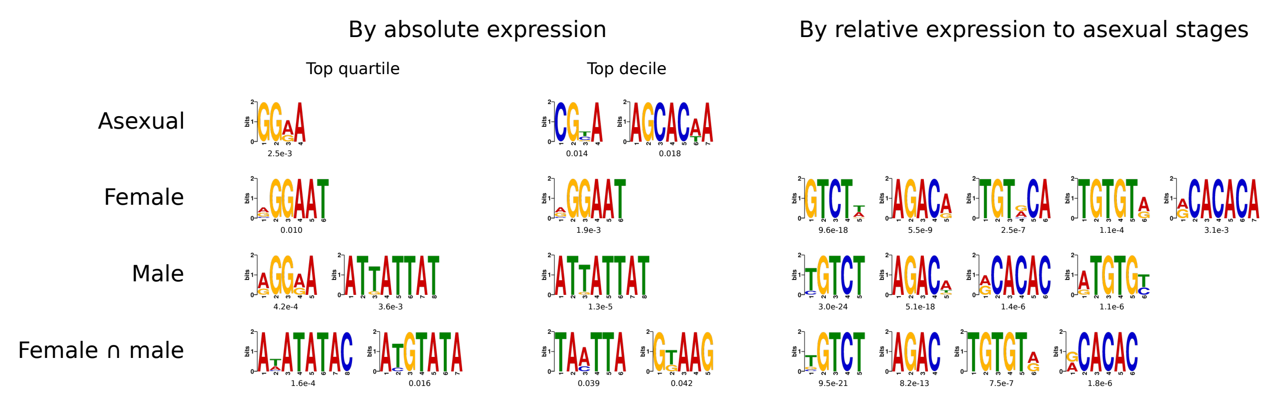

By absolute expression
By relative expression to asexual stages
Top quartile
Top decile
Asexual
2.5e-3
0.014
0.018
Female
0.010
1.9e-3
9.6e-18
5.5e-9
2.5e-7
1.1e-4
3.1e-3
Male
4.2e-4
3.6e-3
1.3e-5
3.0e-24
5.1e-18
1.4e-6
1.1e-6
Female ∩ male
1.6e-4
0.016
0.039
0.042
9.5e-21
8.2e-13
7.5e-7
1.8e-6
