# Supplementary figures and images for: Comparative transcriptomics of female and male gametocytes in Plasmodium berghei and the evolution of sex in alveolates
Source: BMC Genomics. 2017 Sep 18;18:734. doi: 10.1186/s12864-017-4100-0 (PMC5604118; doi:10.1186/s12864-017-4100-0)

Pf male ↑  
1237 total

Pb male ↑  
2497 total

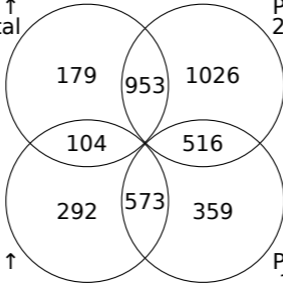

Pb female ↑  
969 total

Pf female ↑  
1448 total

Supplement: Supplementary file 8 — A PDF image comparing the gender-specific transcripts identified in P. berghei compared to those previously identified in P. falciparum. (PDF 11 kb) [file 12864_2017_4100_MOESM8_ESM.pdf]
